# Adults Meeting Fruit and Vegetable Intake Recommendations — United States, 2019

**DOI:** 10.15585/mmwr.mm7101a1

**Published:** 2022-01-07

**Authors:** Seung Hee Lee, Latetia V. Moore, Sohyun Park, Diane M. Harris, Heidi M. Blanck

**Affiliations:** 1 Division of Nutrition, Physical Activity, and Obesity, National Center for Chronic Disease Prevention and Health Promotion, CDC.

The 2020–2025 Dietary Guidelines for Americans* advise incorporating more fruits and vegetables into U.S. residents’ diets as part of healthy dietary patterns. Adults should consume 1.5–2 cup-equivalents of fruits and 2–3 cup-equivalents of vegetables daily.^†^ A healthy diet supports healthy immune function (*1*) and helps to prevent obesity, type 2 diabetes, cardiovascular diseases, and some cancers (*2*); having some of these conditions can predispose persons to more severe illness and death from COVID-19 (*3*). CDC used the most recent 2019 Behavioral Risk Factor Surveillance system (BRFSS) data to estimate the percentage of states’ adult population who met intake recommendations overall and by sociodemographic characteristics for 49 states and the District of Columbia (DC). Overall, 12.3% of adults met fruit recommendations, ranging from 8.4% in West Virginia to 16.1% in Connecticut, and 10.0% met vegetable recommendations, ranging from 5.6% in Kentucky to 16.0% in Vermont. The prevalence of meeting fruit intake recommendations was highest among Hispanic adults (16.4%) and lowest among males (10.1%); meeting vegetable intake recommendations was highest among adults aged ≥51 years (12.5%) and lowest among those living below or close to the poverty level (income to poverty ratio [IPR] <1.25) (6.8%). Additional policies^§^ and programs that will increase access to fruits and vegetables in places where U.S. residents live, learn, work, and play, might increase consumption and improve health.

BRFSS is an annual, state-based, random-digit–dialed telephone survey of health-related behaviors representative of noninstitutionalized adults aged ≥18 years in the United States and participating territories.^¶^ Since 1989, BRFSS has collected information on respondents’ frequency of fruit and vegetable consumption. The current module assesses the number of times per day, week, or month a respondent consumed whole fruit, 100% fruit juice, salads, fried potatoes, other potatoes, and other vegetables during the past 30 days. In 2019, New Jersey data did not meet the minimum requirements for inclusion** and were excluded. Among 418,268 respondents to the current BRFSS, 8,458 residents of Guam and Puerto Rico were excluded, because the scoring algorithms were derived from the National Health and Nutrition Examination Survey (NHANES), which excludes territories, as were 59,589 respondents who did not answer one or more questions in the fruit and vegetable module, 1,347 with implausible reported values of fruit or vegetable intake (>16 times and >23 times per day, respectively), 54,306 who did not report income, and two who did not report race. The resulting analytic sample included 294,566 (70%) participants. Among states included in the analysis, the median state response rate was 49.4% and ranged from 37.3% to 73.1%.^††^

Previously developed scoring algorithms were used to estimate the percentage of each state’s population who met fruit and vegetable intake recommendations. Development of the methodology (*4*) and application of the prediction algorithm have been previously reported.^§§^ Twenty-four–hour dietary recall data from 2013–2016 NHANES were used to fit age- and sex-specific logistic regression models that estimate probabilities of meeting recommendations as functions of reported daily frequency of consumption, race/ethnicity, and IPR, adjusting for day-to-day variation (*4*). Consistent with previous studies (*4*,*5*), analyses accounted for the complex survey design and nonresponse, and balanced repeated replication was used to calculate standard errors and 95% CIs with SAS (version 9.4; SAS Institute). T-tests were used to compare differences by sociodemographic groups with Stata (version 17.0; StataCorp). This activity was reviewed by CDC and was conducted consistent with applicable federal law and CDC policy.^¶¶^

In 2019, the median frequency of reported fruit intake was once per day; this was consistent across all jurisdictions (Table 1). The median frequency of reported vegetable intake was 1.6 times per day, ranging from 1.5 times per day in Louisiana, Mississippi, Nevada, and New Mexico to 1.9 times per day in Maine and Vermont. Among all respondents, 12.3% of adults met fruit intake recommendations, ranging from 8.4% in West Virginia to 16.1% in Connecticut, and 10.0% met vegetable intake recommendations, ranging from 5.6% in Kentucky to 16.0% in Vermont.

**TABLE 1:** State-specific median frequency of fruit and vegetable intake among adults aged ≥18 years and percentage of respondents meeting federal fruit and vegetable recommendations — Behavioral Risk Factor Surveillance System, 49 states* and District of Columbia, 2019

Jurisdiction	Sample size	Median daily intake frequency		% of respondents (95% CI) meeting recommendations	
		Fruit	Vegetable	Fruit	Vegetable
**Overall**	**294,566**	**1.0**	**1.6**	**12.3 (11.2–13.3)**	**10.0 (8.8–11.3)**
Alabama	4,990	1.0	1.6	10.3 (8.7–12.0)	6.7 (5.1–8.3)
Alaska	2,138	1.0	1.7	12.2 (9.5–15.0)	11.4 (8.8–14.0)
Arizona	6,149	1.0	1.6	14.8 (12.6–16.9)	12.8 (10.5–15.0)
Arkansas	3,571	1.0	1.6	11.6 (9.6–13.6)	10.8 (8.7–13.0)
California	8,894	1.0	1.6	13.6 (11.9–15.2)	11.3 (9.6–13.0)
Colorado	6,740	1.0	1.7	12.4 (10.7–14.1)	10.4 (8.7–12.2)
Connecticut	6,228	1.0	1.7	16.1 (14.2–18.0)	14.1 (12.1–16.1)
Delaware	2,684	1.0	1.7	13.4 (10.9–15.8)	9.1 (7.0–11.2)
District of Columbia	1,873	1.0	1.8	14.5 (11.8–17.1)	12.8 (10.3–15.3)
Florida	11,389	1.0	1.7	12.4 (10.6–14.2)	10.5 (8.6–12.5)
Georgia	5,017	1.0	1.7	11.2 (9.3–13.1)	8.9 (6.9–10.8)
Hawaii	6,279	1.0	1.6	11.9 (10.2–13.6)	12.2 (10.3–14.1)
Idaho	3,847	1.0	1.7	10.3 (8.5–12.0)	9.7 (7.6–11.7)
Illinois	4,565	1.0	1.6	12.9 (11.1–14.6)	8.9 (7.2–10.7)
Indiana	5,845	1.0	1.6	13.0 (11.3–14.7)	10.5 (8.7–12.3)
Iowa	7,460	1.0	1.6	10.6 (9.1–12.1)	7.3 (5.8–8.8)
Kansas	8,297	1.0	1.7	10.9 (9.5–12.4)	9.8 (8.1–11.4)
Kentucky	4,743	1.0	1.6	8.8 (7.1–10.4)	5.6 (4.1–7.2)
Louisiana	3,324	1.0	1.5	11.2 (9.2–13.2)	7.3 (5.7–8.9)
Maine	7,902	1.0	1.9	11.9 (10.1–13.7)	10.9 (8.9–12.9)
Maryland	12,464	1.0	1.6	13.5 (11.9–15.2)	9.9 (8.2–11.5)
Massachusetts	5,209	1.0	1.7	13.4 (11.6–15.3)	10.5 (8.6–12.3)
Michigan	8,031	1.0	1.6	11.1 (9.5–12.7)	7.2 (5.7–8.8)
Minnesota	11,732	1.0	1.6	12.7 (11.1–14.2)	8.8 (7.2–10.4)
Mississippi	3,651	1.0	1.5	10.5 (8.6–12.5)	7.7 (6.0–9.4)
Missouri	5,299	1.0	1.6	8.7 (7.2–10.2)	7.4 (5.6–9.2)
Montana	5,073	1.0	1.7	10.0 (8.4–11.5)	9.6 (7.8–11.3)
Nebraska	12,557	1.0	1.6	10.7 (9.2–12.1)	8.0 (6.4–9.6)
Nevada	2,086	1.0	1.5	8.6 (6.9–10.3)	7.4 (5.5–9.3)
New Hampshire	4,043	1.0	1.7	12.9 (11.0–14.8)	12.3 (10.2–14.5)
New Mexico	4,638	1.0	1.5	11.5 (9.7–13.3)	9.2 (7.2–11.1)
New York	9,181	1.0	1.7	15.3 (13.5–17.1)	14.2 (12.3–16.0)
North Carolina	2,971	1.0	1.7	11.0 (9.2–12.8)	9.5 (7.6–11.5)
North Dakota	4,394	1.0	1.6	9.8 (8.0–11.7)	7.3 (5.6–9.1)
Ohio	9,616	1.0	1.6	9.5 (8.0–11.0)	7.4 (5.8–9.0)
Oklahoma	3,958	1.0	1.6	8.7 (7.2–10.2)	6.9 (5.2–8.5)
Oregon	4,303	1.0	1.7	12.9 (11.1–14.7)	12.2 (10.3–14.1)
Pennsylvania	5,150	1.0	1.6	10.6 (9.0–12.3)	8.4 (6.7–10.1)
Rhode Island	4,002	1.0	1.7	14.8 (12.6–17.0)	13.4 (11.1–15.8)
South Carolina	5,050	1.0	1.6	11.9 (10.2–13.7)	10.2 (8.4–12.0)
South Dakota	4,762	1.0	1.6	10.3 (8.1–12.5)	7.4 (5.4–9.5)
Tennessee	4,289	1.0	1.7	11.0 (9.2–12.8)	9.2 (7.3–11.1)
Texas	8,260	1.0	1.6	13.8 (11.7–15.8)	11.9 (9.8–14.0)
Utah	9,011	1.0	1.6	11.5 (10.0–13.1)	8.3 (6.8–9.8)
Vermont	4,530	1.0	1.9	15.3 (13.1–17.6)	16.0 (13.6–18.4)
Virginia	7,268	1.0	1.7	12.2 (10.4–13.9)	9.6 (7.9–11.4)
Washington	9,604	1.0	1.7	12.6 (11.0–14.2)	11.9 (10.1–13.7)
West Virginia	4,117	1.0	1.6	8.4 (6.8–9.9)	6.9 (5.3–8.6)
Wisconsin	3,881	1.0	1.6	11.6 (9.7–13.5)	7.6 (5.9–9.3)
Wyoming	3,501	1.0	1.7	9.4 (7.7–11.2)	8.4 (6.4–10.5)

* New Jersey data did not meet the minimum requirements for inclusion in the 2019 aggregate data set and were excluded.

Fruit intake (Table 2) and vegetable intake (Table 3) varied by sociodemographic characteristics. Overall, a higher proportion of women met both fruit and vegetable recommendations (14.5% and 12.4%, respectively) than did men (10.1% and 7.6%, respectively); a similar pattern was observed across most states. A significantly higher proportion of adults aged ≥51 years (12.5%) met vegetable recommendations compared with younger adults aged 18–30 years (7.1%) and 31–50 years (8.7%). This pattern was also observed in 37 states. A significantly higher proportion of Hispanic adults (16.4%) met fruit intake recommendations compared with those who were non-Hispanic White overall (11.1%); this pattern was observed in 14 states (Table 2). Overall, a significantly lower proportion of non-Hispanic Black adults (6.9%) met vegetable intake recommendations than did their non-Hispanic White counterparts (10.1%); however, this pattern was statistically significant in only three states (California, Massachusetts, and Nevada). Overall, a significantly higher proportion of adults living in households with the highest income category met vegetable intake recommendations (12.2%) than did adults living in middle income households (7.7%) and with the lowest income categories (6.8%); patterns were similar in most states.

**TABLE 2:** State-specific percentage of respondents meeting federal fruit intake recommendations, by sex, age, race/ethnicity, and income-to-poverty ratio — Behavioral Risk Factor Surveillance System, 49 states* and District of Columbia, 2019

Jurisdiction	% (95% CI)										
	Sex		Age group, yrs			Race/Ethnicity^†^			IPR		
	Male	Female (Ref)	18–30	31–50	≥51 (Ref)	Black	Hispanic	White (Ref)	<1.25	1.25–3.49	>3.49 (Ref)
**National**	**10.1^§^ (8.5–11.6)**	**14.5 (13.1–15.8)**	**10.2 (7.7–12.6)**	**13.2 (11.4–14.9)**	**12.6 (11.1–14.1)**	**12.9 (11.1–14.7)**	**16.4^§^ (14.4–18.5)**	**11.1 (10.1–12.2)**	**12.8 (11.2–14.4)**	**10.9^§^ (9.6–12.3)**	**12.9 (11.5–14.2)**
Alabama	9.2 (6.6–11.7)	11.4 (9.4–13.4)	9.5 (4.9–14.2)	11.9 (9.2–14.6)	9.5 (7.6–11.4)	12.9^§^ (9.7–16.0)	17.3 (6.2–28.4)	9.1 (7.5–10.7)	12.0 (8.5–15.4)	8.0^§^ (6.0–10.0)	11.3 (9.1–13.5)
Alaska	9.5^§^ (6.3–12.7)	15.4 (11.2–19.6)	13.6 (4.6–22.6)	10.9 (7.0–14.8)	12.9 (9.8–15.9)	—^¶^	—^¶^	11.2 (8.8–13.6)	16.2 (6.1–26.4)	8.4^§^ (4.7–12.0)	13.4 (10.1–16.7)
Arizona	12.7 (9.7–15.8)	16.8 (13.9–19.7)	13.8 (8.4–19.2)	15.6 (11.8–19.5)	14.5 (11.9–17.1)	19.3 (10.2–28.4)	17.8 (13.3–22.2)	13.1 (11.0–15.3)	19.6 (13.9–25.4)	11.7 (8.8–14.6)	15.1 (12.4–17.7)
Arkansas	10.7 (7.7–13.7)	12.5 (9.9–15.0)	10.3 (5.1–15.5)	12.0 (8.4–15.6)	12.0 (9.7–14.3)	13.1 (7.8–18.4)	18.6 (9.3–27.9)	10.8 (8.9–12.7)	11.1 (7.5–14.7)	10.5 (7.8–13.2)	12.9 (10.0–15.8)
California	11.3^§^ (8.9–13.6)	15.8 (13.7–18.0)	9.5^§^ (6.3–12.8)	15.1 (12.2–18.0)	14.5 (12.2–16.9)	11.2 (7.5–14.8)	16.3^§^ (13.8–18.8)	12.3 (10.5–14.1)	13.9 (11.3–16.5)	13.4 (10.8–15.9)	13.5 (11.4–15.6)
Colorado	10.7^§^ (8.2–13.1)	14.1 (11.9–16.3)	9.8 (6.1–13.6)	14.4 (11.3–17.4)	12.0 (9.8–14.1)	12.7 (7.1–18.2)	15.3 (12.0–18.5)	11.6 (9.9–13.4)	10.8 (7.9–13.7)	10.3^§^ (8.0–12.5)	13.6 (11.5–15.7)
Connecticut	13.6^§^ (10.9–16.3)	18.6 (15.9–21.2)	15.0 (10.0–20.0)	15.8 (12.5–19.1)	16.7 (14.3–19.2)	15.7 (11.0–20.5)	19.8 (15.0–24.7)	15.7 (13.6–17.8)	17.3 (12.8–21.9)	14.4 (11.5–17.2)	16.6 (14.3–18.9)
Delaware	11.3 (7.6–15.0)	15.2 (12.2–18.2)	10.3 (4.2–16.3)	15.3 (10.7–19.9)	13.2 (10.4–16.1)	15.3 (9.7–21.0)	21.8^§^ (13.0–30.6)	11.4 (9.1–13.6)	14.0 (9.5–18.5)	13.6 (9.2–17.9)	13.1 (10.3–15.9)
District of Columbia	11.8 (7.7–16.0)	16.9 (13.7–20.0)	7.2^§^ (2.3–12.0)	17.9 (13.2–22.5)	16.2 (12.6–19.8)	15.0 (11.3–18.8)	15.3 (8.3–22.3)	13.8 (10.4–17.2)	14.8 (8.7–20.9)	14.8 (9.8–19.8)	14.3 (11.3–17.4)
Florida	10.2^§^ (7.7–12.7)	14.5 (12.1–17.0)	10.8 (6.6–15.0)	13.8 (10.4–17.2)	12.1 (9.7–14.4)	14.5 (9.7–19.2)	15.7^§^ (11.6–19.8)	10.7 (9.1–12.4)	13.5 (9.8–17.2)	12.3 (9.5–15.1)	12.1 (9.8–14.4)
Georgia	9.6 (6.7–12.6)	12.7 (10.4–14.9)	9.7 (4.8–14.5)	12.8 (9.5–16.0)	10.6 (8.3–12.9)	11.1 (7.9–14.3)	19.6^§^ (12.6–26.5)	10.0 (8.1–12.0)	12.4 (8.9–15.9)	9.4 (6.7–12.1)	11.8 (9.3–14.3)
Hawaii	9.3^§^ (7.1–11.6)	14.4 (11.9–16.9)	11.3 (7.2–15.4)	13.0 (9.8–16.1)	11.3 (9.0–13.5)	7.6 (1.9–13.4)	16.7 (12.2–21.1)	13.1 (10.7–15.5)	14.7 (10.7–18.6)	11.6 (8.7–14.4)	11.5 (9.5–13.5)
Idaho	8.2^§^ (5.7–10.7)	12.3 (9.9–14.8)	8.1 (3.8–12.4)	10.3 (7.4–13.1)	11.4 (8.9–13.9)	—^¶^	11.8 (6.9–16.6)	10.0 (8.2–11.9)	11.6 (7.5–15.7)	8.5 (6.4–10.7)	11.2 (8.7–13.7)
Illinois	10.1^§^ (7.6–12.6)	15.5 (13.1–17.8)	10.8 (6.6–15.0)	12.5 (9.7–15.4)	14.1 (11.7–16.6)	13.6 (9.6–17.6)	16.3^§^ (12.7–20.0)	11.9 (10.0–13.7)	13.7 (10.1–17.4)	11.2 (8.7–13.7)	13.5 (11.3–15.7)
Indiana	11.1^§^ (8.7–13.6)	14.9 (12.7–17.1)	11.3 (7.3–15.4)	14.3 (11.3–17.2)	12.8 (10.7–14.9)	15.9 (11.4–20.5)	18.7^§^ (12.9–24.6)	12.3 (10.6–14.0)	13.2 (9.9–16.5)	10.8^§^ (8.6–12.9)	14.5 (12.2–16.7)
Iowa	7.7^§^ (5.6–9.7)	13.4 (11.4–15.5)	8.2 (4.5–12.0)	10.8 (8.4–13.2)	11.5 (9.5–13.6)	13.5 (7.0–20.1)	16.7^§^ (11.8–21.6)	10.1 (8.7–11.6)	9.5 (6.7–12.4)	9.3 (7.3–11.3)	11.4 (9.6–13.2)
Kansas	8.7^§^ (6.7–10.7)	13.1 (11.1–15.1)	8.5 (5.2–11.8)	12.1 (9.6–14.6)	11.2 (9.3–13.1)	11.9 (7.7–16.2)	13.2 (9.3–17.2)	10.5 (9.1–12.0)	9.6 (6.6–12.5)	9.1^§^ (7.3–11.0)	12.3 (10.4–14.2)
Kentucky	6.7^§^ (4.5–9.0)	10.8 (8.5–13.1)	8.1 (3.7–12.5)	8.8 (6.1–11.4)	9.1 (6.8–11.3)	14.2 (6.4–22.0)	17.7^§^ (8.3–27.1)	8.0 (6.5–9.5)	10.2 (6.2–14.2)	6.6 (4.4–8.9)	9.6 (7.6–11.7)
Louisiana	10.6 (7.5–13.8)	11.8 (9.5–14.0)	10.8 (5.4–16.2)	11.7 (8.5–14.9)	11.0 (8.7–13.4)	13.8^§^ (9.9–17.6)	18.2 (8.9–27.5)	9.2 (7.4–11.1)	12.7 (9.0–16.5)	9.7 (6.7–12.8)	11.5 (8.9–14.0)
Maine	9.3^§^ (6.7–11.9)	14.5 (12.0–17.0)	10.3 (4.8–15.7)	12.9 (9.8–16.1)	11.9 (9.8–14.0)	—^¶^	27.5 (9.9–45.1)	11.7 (10.0–13.5)	8.0^§^ (5.3–10.7)	9.3^§^ (6.9–11.6)	14.5 (12.1–17.0)
Maryland	10.8^§^ (8.4–13.1)	16.1 (13.9–18.3)	12.4 (8.1–16.7)	13.8 (11.1–16.5)	13.8 (11.7–16.0)	13.9 (11.1–16.7)	18.0^§^ (13.3–22.7)	12.8 (11.1–14.6)	14.3 (10.7–17.8)	11.9 (9.5–14.3)	14.1 (12.1–16.1)
Massachusetts	11.9 (9.2–14.7)	14.9 (12.5–17.3)	10.6 (6.2–15.0)	15.6 (12.2–18.9)	13.1 (10.7–15.5)	12.0 (7.0–17.0)	15.6 (11.2–20.0)	13.5 (11.5–15.4)	12.8 (8.8–16.7)	12.2 (9.4–15.0)	14.0 (11.8–16.2)
Michigan	8.0^§^ (5.8–10.1)	14.2 (12.0–16.5)	7.8^§^ (4.2–11.4)	11.5 (8.8–14.2)	12.2 (10.1–14.4)	13.7 (9.8–17.5)	11.0 (6.8–15.1)	10.8 (9.2–12.4)	11.1 (8.0–14.2)	9.2^§^ (7.2–11.2)	12.1 (10.1–14.1)
Minnesota	9.7^§^ (7.7–11.8)	15.6 (13.4–17.8)	9.4^§^ (5.9–12.8)	12.3 (9.8–14.8)	14.3 (12.1–16.6)	13.1 (8.7–17.6)	15.4 (11.1–19.7)	12.6 (11.0–14.1)	14.2 (10.7–17.6)	9.8^§^ (7.9–11.6)	13.6 (11.8–15.5)
Mississippi	9.2 (6.0–12.5)	11.7 (9.5–13.9)	12.0 (5.6–18.4)	11.2 (8.4–14.0)	9.3 (7.2–11.3)	12.3^§^ (9.1–15.6)	—^¶^	8.3 (6.5–10.0)	9.4 (6.6–12.3)	9.7 (7.0–12.5)	11.8 (8.9–14.8)
Missouri	6.6^§^ (4.5–8.7)	10.7 (8.7–12.8)	6.0 (2.7–9.4)	9.5 (7.0–12.1)	9.3 (7.3–11.3)	11.1 (7.2–14.9)	10.0 (4.1–15.9)	8.2 (6.7–9.6)	8.0 (5.1–10.8)	6.7^§^ (4.9–8.5)	10.1 (8.1–12.1)
Montana	8.1^§^ (5.9–10.2)	12.0 (9.8–14.2)	6.2^§^ (2.8–9.7)	10.9 (8.1–13.7)	11.0 (8.9–13.0)	—^¶^	12.8 (5.5–20.0)	9.7 (8.2–11.3)	8.7 (5.9–11.4)	8.3^§^ (6.3–10.4)	11.6 (9.5–13.7)
Nebraska	8.3^§^ (6.2–10.4)	13.1 (11.1–15.1)	9.2 (5.6–12.9)	10.6 (8.2–13.1)	11.4 (9.4–13.3)	13.5 (7.5–19.5)	14.9^§^ (11.4–18.4)	10.2 (8.8–11.7)	10.3 (7.7–12.8)	9.3 (7.3–11.2)	11.6 (9.7–13.5)
Nevada	7.6 (5.1–10.1)	9.7 (7.4–12.0)	5.5 (2.1–9.0)	9.8 (6.7–12.9)	8.9 (6.4–11.4)	8.4 (4.0–12.9)	11.7 (7.8–15.5)	8.1 (6.2–10.0)	9.9 (5.7–14.1)	8.2 (5.5–10.9)	8.5 (6.4–10.6)
New Hampshire	10.1^§^ (7.5–12.7)	15.7 (12.9–18.5)	10.8 (5.4–16.1)	11.7 (8.5–15.0)	14.4 (11.9–16.9)	—^¶^	—^¶^	12.8 (10.9–14.7)	8.5^§^ (5.3–11.7)	12.4 (9.5–15.2)	13.8 (11.4–16.2)
New Mexico	8.7^§^ (6.4–11.1)	14.3 (11.8–16.9)	7.6 (3.8–11.5)	13.5 (10.1–16.9)	11.9 (9.6–14.1)	12.6 (0.5–24.7)	13.5 (10.7–16.3)	8.9 (7.1–10.7)	13.8 (10.3–17.2)	10.0 (7.4–12.7)	11.6 (9.2–13.9)
New York	13.5^§^ (10.8–16.2)	17.1 (14.8–19.4)	12.8 (8.5–17.1)	15.6 (12.6–18.7)	16.3 (13.8–18.7)	15.9 (12.0–19.7)	18.6 (14.9–22.3)	14.1 (12.2–16.0)	17.7 (13.8–21.6)	14.9 (12.2–17.6)	14.8 (12.6–17.0)
North Carolina	9.3 (6.6–12.0)	12.7 (10.3–15.0)	10.2 (5.4–15.0)	12.5 (9.3–15.6)	10.2 (8.0–12.5)	10.1 (7.0–13.1)	13.4 (8.7–18.1)	10.8 (8.8–12.8)	8.1^§^ (5.3–10.9)	10.4 (7.3–13.5)	12.1 (9.9–14.4)
North Dakota	7.3^§^ (4.9–9.6)	12.8 (10.0–15.5)	8.4 (3.9–12.9)	9.5 (6.5–12.6)	10.9 (8.6–13.2)	—^¶^	16.5 (6.3–26.8)	9.3 (7.6–11.0)	11.5 (6.1–16.8)	7.0^§^ (4.9–9.2)	11.0 (8.7–13.3)
Ohio	7.6^§^ (5.4–9.7)	11.3 (9.4–13.3)	7.6 (3.6–11.6)	10.1 (7.6–12.6)	9.9 (8.1–11.7)	11.0 (7.2–14.7)	20.4^§^ (10.6–30.2)	8.7 (7.3–10.2)	7.6^§^ (5.3–10.0)	8.4 (6.4–10.5)	10.8 (8.8–12.8)
Oklahoma	7.0^§^ (4.8–9.2)	10.2 (8.2–12.2)	8.8 (4.6–13.0)	8.8 (6.3–11.3)	8.5 (6.6–10.4)	8.5 (4.0–12.9)	12.9 (7.6–18.2)	8.3 (6.8–9.9)	7.9 (5.0–10.8)	7.5 (5.4–9.6)	9.8 (7.7–11.9)
Oregon	10.1^§^ (7.8–12.5)	15.6 (13.1–18.2)	10.4 (6.2–14.6)	13.2 (10.3–16.2)	13.7 (11.1–16.3)	—^¶^	13.3 (9.2–17.4)	13.3 (11.4–15.2)	11.6 (7.9–15.2)	11.4 (8.9–13.9)	14.1 (11.8–16.4)
Pennsylvania	8.1^§^ (5.9–10.2)	13.1 (10.8–15.5)	8.3 (4.4–12.3)	12.1 (9.0–15.1)	10.5 (8.4–12.6)	13.7 (9.5–17.8)	15.5 (9.6–21.4)	9.8 (8.2–11.5)	10.4 (6.5–14.3)	8.5^§^ (6.3–10.6)	11.8 (9.7–13.8)
Rhode Island	12.8 (9.7–16.0)	16.7 (13.8–19.6)	10.9 (5.8–16.1)	16.7 (12.5–20.9)	15.2 (12.6–17.8)	15.5 (8.5–22.6)	16.7 (10.7–22.7)	14.1 (11.9–16.3)	14.6 (10.1–19.0)	12.6 (9.4–15.8)	15.8 (13.0–18.5)
South Carolina	10.3 (7.7–12.9)	13.5 (11.1–15.9)	10.2 (6.0–14.5)	13.5 (10.1–16.9)	11.6 (9.5–13.8)	15.1^§^ (11.5–18.7)	16.9 (6.6–27.2)	10.4 (8.7–12.1)	12.7 (9.1–16.3)	11.0 (8.2–13.8)	12.3 (10.0–14.5)
South Dakota	8.3 (5.1–11.4)	12.5 (9.5–15.4)	7.6 (2.2–13.0)	10.6 (6.9–14.2)	11.4 (8.5–14.3)	—^¶^	23.1 (7.5–38.8)	9.7 (7.7–11.8)	12.1 (5.9–18.4)	8.1 (5.2–11.0)	11.3 (8.5–14.1)
Tennessee	9.9 (7.1–12.7)	12.2 (10.0–14.4)	10.1 (5.3–14.9)	10.6 (7.7–13.5)	11.8 (9.5–14.2)	11.6 (7.8–15.4)	15.3 (6.1–24.5)	10.7 (8.8–12.6)	10.5 (7.4–13.5)	9.5 (6.9–12.1)	12.3 (9.7–14.8)
Texas	11.5^§^ (8.6–14.4)	16.2 (13.4–18.9)	13 (8.1–17.9)	14.5 (11.2–17.8)	13.6 (10.8–16.5)	12.8 (8.1–17.4)	17.5^§^ (13.7–21.4)	11.0 (9.1–12.9)	15.1 (10.8–19.4)	13.2 (9.7–16.7)	13.7 (11.2–16.2)
Utah	8.5^§^ (6.4–10.7)	14.6 (12.6–16.7)	8.0^§^ (4.6–11.3)	12.5 (10.0–15.0)	12.9 (10.7–15.1)	8.6 (1.6–15.6)	15.4^§^ (11.7–19.1)	10.9 (9.4–12.5)	10.2 (7.0–13.4)	10.1 (8.1–12.2)	12.5 (10.6–14.4)
Vermont	11.5^§^ (8.6–14.3)	19.1 (15.8–22.3)	10.8 (5.2–16.5)	17.0 (12.8–21.3)	16.0 (13.4–18.7)	—^¶^	13.9 (3.4–24.5)	15.4 (13.2–17.6)	11.1^§^ (7.0–15.3)	12.2^§^ (9.2–15.1)	18.0 (15.0–21.0)
Virginia	9.1^§^ (6.7–11.5)	15.2 (12.8–17.6)	11.2 (6.5–15.8)	13.0 (10.0–15.9)	12.1 (9.9–14.3)	14.0 (10.6–17.3)	16.6^§^ (11.6–21.7)	10.9 (9.2–12.6)	11.5 (8.2–14.8)	9.5^§^ (7.3–11.8)	13.6 (11.3–15.9)
Washington	10.1^§^ (7.9–12.3)	15.1 (12.9–17.3)	9.7 (6.0–13.3)	13.2 (10.5–16.0)	13.4 (11.2–15.6)	14.2 (8.1–20.4)	16.1 (12.3–19.9)	12.4 (10.8–14.1)	12.0 (8.9–15.2)	11.6 (9.4–13.9)	13.0 (11.1–14.9)
West Virginia	6.1^§^ (4.2–8.1)	10.6 (8.3–12.9)	8.5 (3.8–13.3)	8.7 (6.0–11.4)	8.0 (6.4–9.7)	13.4 (3.1–23.7)	—^¶^	8.1 (6.6–9.6)	8.3 (5.4–11.2)	6.9 (5.0–8.9)	9.7 (7.5–12.0)
Wisconsin	7.7^§^ (5.4–9.9)	15.5 (12.7–18.4)	7.9 (3.3–12.5)	12.2 (8.9–15.5)	12.7 (10.2–15.2)	7.9 (2.3–13.4)	13.5 (6.4–20.6)	11.4 (9.6–13.3)	10.0 (6.0–13.9)	10.8 (7.9–13.6)	12.4 (10.1–14.6)
Wyoming	7.2^§^ (4.7–9.6)	11.8 (9.4–14.2)	6.8 (2.4–11.2)	9.3 (6.3–12.4)	10.7 (8.4–13.0)	—^¶^	12.1 (6.4–17.8)	9.0 (7.3–10.7)	8.9 (5.0–12.7)	8.0 (5.7–10.3)	10.4 (8.1–12.7)

**Abbreviations:** IPR = income-to-poverty ratio; Ref = referent group.

* New Jersey data did not meet the minimum requirements for inclusion in the 2019 aggregate data set and were excluded.

^†^ Black and White persons are non-Hispanic; Hispanic persons could be of any race. Other racial/ethnic groups were not reported because of small sample sizes but were included in overall estimates and estimates by other demographic characteristics.

^§^ p<0.05 for t-test comparing differences by demographic groups to the Ref.

^¶^ Sample sizes <50 were considered unstable and were not reported.

**TABLE 3:** State-specific percentage of respondents meeting federal vegetable intake recommendations by sex, age, race/ethnicity, and income-to-poverty ratio — Behavioral Risk Factor Surveillance System, 49 states* and District of Columbia, 2019

Jurisdiction	% (95% CI)										
	Sex		Age group, yrs			Race/Ethnicity^†^			IPR		
	Men	Women (Ref)	18–30	31–50	≥51 (Ref)	Black	Hispanic	White (Ref)	<1.25	1.25–3.49	>3.49 (Ref)
**National**	**7.6^§^ (5.8–9.4)**	**12.4 (10.6–14.3)**	**7.1^§^ (5.0–9.3)**	**8.7 (6.5–10.8)**	**12.5 (10.3–14.6)**	**6.9^§^ (5.2–8.6)**	**11.0 (9.3–12.6)**	**10.1 (8.4–11.8)**	**6.8^§^ (5.0–8.5)**	**7.7^§^ (5.9–9.4)**	**12.2 (10.5–14.0)**
Alabama	5.6 (3.5–7.7)	7.7 (5.6–9.8)	5.3 (3.0–7.5)	6.2 (4.0–8.5)	7.6 (5.4–9.9)	5.0 (1.7–8.3)	10.5 (7.2–13.8)	7.1 (3.8–10.4)	4.4^§^ (2.0–6.8)	4.3^§^ (1.9–6.6)	9.4 (7.0–11.7)
Alaska	9.6 (6.1–13.0)	13.5 (10.0–16.9)	10.3 (6.4–14.2)	8.7 (4.8–12.6)	14.6 (10.7–18.4)	—^¶^	—^¶^	11.5 (6.9–16.1)	9.5 (6.2–12.8)	9.1 (5.8–12.4)	12.9 (9.6–16.2)
Arizona	10.4^§^ (7.2–13.5)	15.2 (12.1–18.3)	11.2 (8.0–14.3)	11.6 (8.5–14.8)	14.3 (11.2–17.4)	8.4 (3.9–13.0)	14.5 (9.9–19.0)	12.5 (7.9–17.1)	8.9^§^ (5.7–12.2)	10.9 (7.7–14.1)	15.2 (11.9–18.4)
Arkansas	9.0 (6.0–12.1)	12.6 (9.5–15.6)	10.6 (7.8–13.4)	9.5 (6.7–12.3)	12.0 (9.2–14.8)	11.2 (0–23.3)	12.1 (0–24.2)	10.4 (0–22.6)	9.6 (6.6–12.6)	9.5 (6.4–12.5)	12.7 (9.7–15.7)
California	7.9^§^ (5.3–10.5)	14.7 (12.1–17.3)	7.9^§^ (4.9–10.8)	9.7 (6.7–12.6)	14.7 (11.7–17.6)	6.7^§^ (3.9–9.5)	10.1 (7.3–12.9)	12.0 (9.1–14.8)	7.3^§^ (5.0–9.7)	9.7^§^ (7.3–12.0)	13.6 (11.3–16.0)
Colorado	7.9^§^ (5.2–10.6)	13.0 (10.3–15.7)	8.1^§^ (5.1–11.1)	8.7 (5.7–11.7)	13.2 (10.2–16.2)	6.7 (3.0–10.4)	9.5 (5.8–13.2)	10.7 (7.0–14.4)	6.0^§^ (3.7–8.3)	8.2^§^ (5.9–10.5)	12.1 (9.8–14.4)
Connecticut	11.0^§^ (8.0–13.9)	17.2 (14.2–20.1)	10.2^§^ (7.2–13.2)	11.7 (8.7–14.7)	17.1 (14.2–20.1)	9.7 (5.6–13.7)	13.4 (9.4–17.5)	14.8 (10.7–18.8)	9.7^§^ (7.1–12.3)	11.0^§^ (8.4–13.6)	16.2 (13.6–18.8)
Delaware	6.6^§^ (3.6–9.7)	11.3 (8.3–14.4)	4.2^§^ (1.0–7.5)	7.9 (4.6–11.2)	11.8 (8.5–15.1)	5.4 (0–12.3)	7.4 (0.6–14.3)	10.0 (3.1–16.9)	4.7^§^ (1.7–7.8)	5.4^§^ (2.3–8.5)	11.6 (8.5–14.7)
District of Columbia	10.5 (7.1–14.0)	14.9 (11.5–18.3)	10.6 (6.9–14.2)	12.4 (8.8–16.1)	15.1 (11.4–18.7)	7.9 (1.3–14.4)	12.2 (5.7–18.8)	15.9 (9.4–22.4)	4.7^§^ (1.4–8.0)	7.8^§^ (4.5–11.0)	15.8 (12.5–19.1)
Florida	8.1^§^ (5.3–10.9)	12.9 (10.1–15.7)	8.5 (5.5–11.6)	8.5 (5.4–11.6)	12.5 (9.5–15.6)	8.5 (4.6–12.4)	11.2 (7.3–15.1)	10.7 (6.8–14.6)	7.0^§^ (3.9–10.1)	7.9^§^ (4.8–11.0)	13.5 (10.4–16.6)
Georgia	6.4^§^ (3.7–9.1)	11.2 (8.5–13.9)	7.1 (4.1–10.1)	7.3 (4.4–10.3)	11.0 (8.0–14.0)	7.1 (2.3–11.9)	6.6 (1.7–11.4)	10.0 (5.1–14.8)	6.7 (3.8–9.6)	6.9 (4.0–9.8)	10.7 (7.8–13.7)
Hawaii	9.7^§^ (6.9–12.6)	14.6 (11.8–17.5)	8.7^§^ (5.8–11.6)	11.4 (8.5–14.3)	14.0 (11.1–16.9)	9.2 (7.2–11.2)	17.5^§^ (15.5–19.5)	13.4 (11.4–15.4)	11.2 (8.7–13.7)	9.4^§^ (6.9–12.0)	13.6 (11.1–16.2)
Idaho	7.4^§^ (4.5–10.4)	11.9 (9.0–14.9)	6.9^§^ (3.6–10.2)	7.4^§^ (4.1–10.7)	12.9 (9.6–16.3)	—^¶^	11.4 (7.6–15.2)	9.7 (5.9–13.5)	6.8^§^ (4.0–9.7)	7.4^§^ (4.6–10.3)	12.1 (9.2–15.0)
Illinois	6.2^§^ (3.8–8.7)	11.5 (9.0–14.0)	5.6^§^ (2.8–8.5)	7.7 (4.9–10.6)	11.4 (8.6–14.3)	5.5 (1.9–9.1)	8.6 (5.0–12.2)	9.5 (6.0–13.1)	6.3^§^ (3.9–8.7)	5.2^§^ (2.8–7.6)	11.4 (8.9–13.8)
Indiana	8.3^§^ (5.7–10.9)	12.8 (10.2–15.3)	8.1^§^ (5.3–10.9)	9.7 (6.9–12.5)	12.4 (9.6–15.1)	6.6 (0.7–12.5)	10.8 (4.9–16.7)	10.8 (4.9–16.7)	8.6^§^ (5.9–11.2)	7.7^§^ (5.1–10.4)	13.0 (10.3–15.6)
Iowa	5.0^§^ (2.7–7.4)	9.6 (7.2–11.9)	5.0^§^ (2.4–7.5)	5.5^§^ (3.0–8.1)	9.7 (7.2–12.3)	7.2 (3.7–10.7)	8.5 (4.9–12.0)	7.3 (3.8–10.8)	4.8^§^ (2.8–6.9)	5.4^§^ (3.4–7.5)	8.7 (6.7–10.8)
Kansas	7.1^§^ (4.5–9.6)	12.4 (9.9–15.0)	6.6^§^ (3.9–9.3)	8.5 (5.8–11.2)	12.3 (9.6–15.0)	5.8 (2.5–9.2)	9.2 (5.9–12.6)	10.0 (6.6–13.3)	6.1^§^ (3.7–8.4)	8.0^§^ (5.6–10.3)	11.7 (9.3–14.0)
Kentucky	4.0^§^ (1.6–6.3)	7.3 (5.0–9.6)	4.0 (1.4–6.6)	4.5 (1.9–7.1)	7.3 (4.7–9.9)	2.2 (0–5.9)	8.1 (4.4–11.8)	5.9 (2.2–9.6)	3.0^§^ (0.8–5.2)	3.6^§^ (1.4–5.9)	7.8 (5.5–10.0)
Louisiana	5.9 (3.7–8.1)	8.7 (6.5–10.9)	4.4^§^ (1.8–7.0)	6.1^§^ (3.5–8.7)	9.8 (7.2–12.4)	5.0 (0.8–9.3)	11.0 (6.8–15.3)	7.8 (3.6–12.1)	5.0^§^ (2.5–7.4)	5.3^§^ (2.9–7.8)	9.6 (7.2–12.1)
Maine	7.8^§^ (5.1–10.5)	14.0 (11.2–16.7)	8.3^§^ (5.6–11.1)	9.0^§^ (6.2–11.7)	13.0 (10.3–15.8)	—^¶^	18.6^§^ (14.6–22.6)	10.9 (6.8–14.9)	6.6^§^ (3.6–9.6)	7.3^§^ (4.3–10.3)	14.2 (11.2–17.2)
Maryland	6.6^§^ (4.1–9.2)	12.9 (10.3–15.4)	6.5^§^ (3.8–9.3)	7.3^§^ (4.5–10.0)	13.2 (10.5–15.9)	7.5 (3.9–11.1)	12.7 (9.0–16.3)	10.1 (6.5–13.8)	6.2^§^ (4.0–8.4)	6.8^§^ (4.6–9.0)	11.8 (9.6–14.0)
Massachusetts	8.1^§^ (5.3–10.8)	12.7 (10.0–15.4)	6.2^§^ (3.1–9.2)	8.6^§^ (5.6–11.6)	13.7 (10.7–16.7)	6.0^§^ (3.1–8.9)	8.9 (5.9–11.8)	11.1 (8.2–14.0)	5.6^§^ (3.2–8.0)	6.9^§^ (4.5–9.3)	12.5 (10.1–14.9)
Michigan	4.9^§^ (2.5–7.2)	9.6 (7.3–12.0)	4.8^§^ (2.3–7.3)	5.4^§^ (2.9–7.9)	9.5 (7.0–12.0)	4.8 (2.1–7.6)	12.7^§^ (9.9–15.4)	7.2 (4.5–10.0)	4.5^§^ (2.4–6.7)	5.0^§^ (2.9–7.2)	9.1 (7.0–11.3)
Minnesota	6.1^§^ (3.7–8.6)	11.5 (9.1–13.9)	5.7^§^ (3.0–8.4)	6.8^§^ (4.1–9.5)	11.7 (9.0–14.4)	5.5 (2.1–9.0)	8.6 (5.1–12.1)	8.8 (5.3–12.3)	6.7^§^ (4.7–8.8)	5.9^§^ (3.8–7.9)	10.3 (8.2–12.4)
Mississippi	6.3 (4.0–8.6)	8.9 (6.6–11.2)	4.9^§^ (2.2–7.5)	7.2 (4.5–9.8)	9.5 (6.8–12.1)	5.8 (1.4–10.2)	—^¶^	8.9 (4.4–13.3)	3.5^§^ (0.7–6.2)	6.6^§^ (3.9–9.4)	10.7 (8.0–13.5)
Missouri	5.5^§^ (3.0–8.0)	9.3 (6.8–11.8)	4.9^§^ (2.2–7.6)	6.4 (3.7–9.1)	9.4 (6.6–12.1)	4.5 (0–9.5)	9.2 (4.3–14.2)	7.6 (2.7–12.6)	4.2^§^ (1.7–6.8)	5.7^§^ (3.1–8.2)	9.3 (6.8–11.8)
Montana	8.5 (6.0–11.1)	10.6 (8.1–13.2)	7.7 (5.1–10.3)	8.9 (6.3–11.5)	10.7 (8.1–13.4)	—^¶^	16.6^§^ (13.4–19.9)	9.5 (6.2–12.7)	7.2^§^ (4.7–9.8)	7.2^§^ (4.6–9.7)	12.0 (9.4–14.5)
Nebraska	5.8^§^ (3.4–8.1)	10.2 (7.9–12.6)	5.9^§^ (3.4–8.5)	6.2^§^ (3.6–8.8)	10.3 (7.7–12.9)	5.8 (2.1–9.5)	7.1 (3.4–10.8)	8.1 (4.4–11.8)	5.1^§^ (2.8–7.4)	5.3^§^ (3.0–7.6)	10.3 (8.0–12.7)
Nevada	5.6^§^ (2.8–8.3)	9.5 (6.7–12.2)	5.8 (2.9–8.8)	5.9 (3.0–8.9)	9.4 (6.4–12.3)	5.4^§^ (3.2–7.5)	6.4 (4.3–8.6)	9.0 (6.8–11.1)	4.2^§^ (1.5–6.8)	5.5^§^ (2.9–8.1)	9.5 (6.8–12.1)
New Hampshire	9.6^§^ (6.4–12.9)	15.1 (11.8–18.3)	10.3^§^ (7.1–13.5)	9.2^§^ (6.1–12.4)	15.0 (11.8–18.2)	—^¶^	—^¶^	12.0 (7.1–17.0)	6.5^§^ (3.5–9.4)	8.4^§^ (5.4–11.3)	14.9 (12.0–17.8)
New Mexico	6.8^§^ (4.1–9.6)	11.5 (8.7–14.3)	4.8^§^ (1.9–7.6)	8.6 (5.8–11.5)	11.6 (8.7–14.5)	4.3 (0.1–8.4)	9.7 (5.6–13.9)	8.4 (4.2–12.6)	8.1 (5.3–10.9)	7.2^§^ (4.4–10.0)	11.3 (8.4–14.1)
New York	10.9^§^ (8.1–13.7)	17.4 (14.6–20.2)	8.9^§^ (5.9–11.8)	13.3 (10.4–16.3)	17.3 (14.3–20.2)	10.4 (5.8–15.0)	13.4 (8.8–18.0)	14.3 (9.7–18.9)	11.2^§^ (8.7–13.7)	12.8 (10.3–15.4)	15.8 (13.2–18.3)
North Carolina	7.6 (4.7–10.5)	11.4 (8.6–14.3)	7.4^§^ (4.3–10.6)	7.6^§^ (4.5–10.8)	12.1 (9.0–15.2)	8.0 (4.5–11.6)	7.1 (3.5–10.6)	10.3 (6.7–13.9)	4.8^§^ (2.0–7.5)	7.0^§^ (4.2–9.8)	12.2 (9.5–15.0)
North Dakota	5.4^§^ (2.6–8.2)	9.5 (6.7–12.4)	6.0 (3.3–8.7)	5.5^§^ (2.8–8.1)	9.6 (6.9–12.3)	—^¶^	8.6 (3.6–13.7)	6.9 (1.9–12.0)	5.5^§^ (3.1–7.9)	4.2^§^ (1.8–6.6)	9.2 (6.8–11.6)
Ohio	5.7^§^ (3.5–8.0)	9.1 (6.8–11.3)	4.8^§^ (2.4–7.2)	6.6 (4.2–9.0)	9.2 (6.8–11.6)	5.6 (2.2–9.0)	13.0^§^ (9.6–16.4)	7.3 (3.9–10.7)	4.5^§^ (2.1–6.8)	5.4^§^ (3.1–7.7)	9.7 (7.3–12.0)
Oklahoma	5.7 (3.5–8.0)	7.9 (5.6–10.1)	4.6^§^ (2.0–7.2)	6.1 (3.5–8.7)	8.5 (5.9–11.1)	5.4 (2.5–8.3)	7.4 (4.5–10.3)	7.0 (4.1–9.9)	3.4^§^ (0.8–6.0)	4.7^§^ (2.2–7.3)	9.6 (7.0–12.1)
Oregon	9.4^§^ (6.5–12.3)	14.9 (12.0–17.9)	9.1^§^ (6.0–12.2)	10.4^§^ (7.3–13.5)	14.8 (11.8–17.9)	—^¶^	8.9 (3.8–13.9)	12.3 (7.2–17.3)	7.7^§^ (5.1–10.4)	10.0^§^ (7.4–12.7)	14.4 (11.8–17.1)
Pennsylvania	5.8^§^ (3.2–8.4)	10.9 (8.3–13.6)	5.4^§^ (2.7–8.1)	7.1 (4.4–9.7)	10.5 (7.8–13.1)	5.7 (2.3–9.2)	10.6 (7.1–14.0)	8.5 (5.0–11.9)	6.5^§^ (4.2–8.8)	4.7^§^ (2.4–7.0)	10.7 (8.4–13.0)
Rhode Island	9.3^§^ (5.7–13.0)	17.4 (13.7–21.0)	9.9^§^ (6.9–13.0)	12.0 (8.9–15.1)	15.8 (12.7–18.9)	8.6 (1.4–15.8)	8.7 (1.4–15.9)	13.9 (6.7–21.2)	8.2^§^ (5.3–11.0)	12.6 (9.7–15.5)	14.9 (12.0–17.8)
South Carolina	8.2^§^ (5.6–10.8)	12.0 (9.4–14.7)	8.5 (5.8–11.2)	8.3^§^ (5.5–11.0)	12.3 (9.6–15.0)	7.6 (2.3–13.0)	13.6 (8.2–18.9)	10.6 (5.2–15.9)	6.2^§^ (3.6–8.8)	7.6^§^ (5.0–10.3)	12.9 (10.2–15.5)
South Dakota	5.9 (2.9–8.8)	9.1 (6.2–12.1)	4.6^§^ (1.4–7.9)	6.5 (3.2–9.8)	9.6 (6.3–12.8)	—^¶^	14.5 (8.9–20.1)	7.0 (1.4–12.6)	7.2 (4.4–9.9)	4.5^§^ (1.7–7.2)	9.3 (6.5–12.0)
Tennessee	7.6 (4.9–10.2)	10.8 (8.2–13.5)	6.9^§^ (4.0–9.8)	7.6 (4.7–10.5)	11.4 (8.5–14.3)	5.8 (0.1–11.5)	11.5 (5.8–17.2)	9.6 (3.9–15.3)	6.7^§^ (3.9–9.5)	6.8^§^ (4.0–9.5)	11.7 (8.9–14.5)
Texas	10.2 (7.5–12.9)	13.6 (11.0–16.3)	8.8^§^ (5.2–12.5)	11.3 (7.7–14.9)	14.4 (10.8–18.0)	7.6 (2.8–12.4)	12.7 (7.9–17.5)	12.7 (7.9–17.5)	7.8^§^ (4.9–10.7)	10.6 (7.7–13.5)	14.1 (11.2–17.0)
Utah	6.3^§^ (4.0–8.5)	10.4 (8.1–12.7)	5.1^§^ (2.2–8.1)	7.3^§^ (4.4–10.2)	11.8 (8.9–14.8)	5.2 (2.1–8.4)	9.0 (5.9–12.2)	8.2 (5.1–11.4)	5.4^§^ (3.4–7.4)	5.9^§^ (3.9–8.0)	10.0 (8.0–12.1)
Vermont	10.8^§^ (7.1–14.5)	21.0 (17.3–24.7)	13.3^§^ (10.1–16.6)	13.7^§^ (10.4–17.0)	18.5 (15.2–21.7)	—^¶^	7.9 (0.6–15.2)	16.2 (8.9–23.5)	11.6^§^ (8.3–14.8)	12.9^§^ (9.6–16.2)	18.6 (15.3–21.9)
Virginia	7.1^§^ (4.4–9.7)	12.1 (9.5–14.8)	5.9^§^ (2.9–8.9)	8.0^§^ (5.0–11.0)	12.7 (9.7–15.7)	6.9 (3.8–10.0)	10.5 (7.4–13.6)	10.4 (7.3–13.5)	4.3^§^ (1.9–6.8)	6.6^§^ (4.1–9.0)	12.2 (9.8–14.7)
Washington	9.2^§^ (6.5–11.9)	14.6 (11.9–17.3)	6.7^§^ (3.8–9.7)	11.1 (8.1–14.0)	14.9 (11.9–17.9)	10.3 (7.3–13.2)	13.5 (10.5–16.4)	11.9 (8.9–14.8)	8.9^§^ (6.6–11.2)	9.4^§^ (7.1–11.7)	13.3 (11.0–15.6)
West Virginia	4.9^§^ (2.5–7.2)	9.0 (6.7–11.4)	3.5^§^ (1.3−5.7)	4.7^§^ (2.4−6.9)	7.5 (5.3−9.7)	4.9 (2.0–7.9)	—^¶^	7.0 (4.0–10.0)	4.8^§^ (2.2–7.4)	5.1^§^ (2.5–7.6)	9.7 (7.2–12.3)
Wisconsin	5.0^§^ (2.5–7.6)	10.1 (7.6–12.7)	5.7^§^ (3.1−8.2)	7.2 (4.7−9.8)	9.1 (6.6−11.7)	5.5 (1.1–9.9)	8.4 (4.0–12.8)	7.6 (3.2–12.0)	6.0 (3.6–8.5)	5.0^§^ (2.6–7.5)	9.3 (6.8–11.7)
Wyoming	5.3^§^ (2.1–8.5)	11.8 (8.6–14.9)	7.8^§^ (4.9−10.7)	7.6^§^ (4.7−10.5)	11.0 (8.1−13.9)	—^¶^	9.0 (3.9–14.1)	8.4 (3.3–13.5)	4.1^§^ (1.3–7.0)	6.1^§^ (3.2–8.9)	10.6 (7.7–13.4)

**Abbreviations:** IPR = income-to-poverty ratio; Ref = referent group.

* New Jersey data did not meet the minimum requirements for inclusion in the 2019 aggregate data set and were excluded.

^†^ Black and White persons are non-Hispanic; Hispanic persons could be of any race. Other racial/ethnic group not reported because of small sample sizes but were included in overall estimates and estimates by other demographic characteristics.

^§^ p<0.05 for t-test comparing differences by demographic groups to the Ref.

^¶^ Sample sizes <50 were considered unstable and were not reported.

## Discussion

In 2019, fruit and vegetable intake among U.S. adults remained low, with only approximately one in 10 adults meeting either recommendation; differences were found by state, age, sex, race/ethnicity, and household income. Consistent with previous analyses of BRFSS data (*4*,*5*), a higher percentage of women than men met recommendations for fruit and vegetable intake, and larger disparities were observed in vegetable intake than fruit intake by age groups and household income. Results were also consistent with earlier findings (*5*) that higher percentages of Hispanic than non-Hispanic White adults met fruit intake recommendations while lower percentages of non-Hispanic Black than non-Hispanic White adults met vegetable intake recommendations. In 2015, intake was also low: 12.2% of respondents met fruit intake recommendations and 9.3% met vegetable intake recommendations (*5*); however, direct comparisons between current findings to those of 2015 cannot be made because of changes in methodology.***

Perceived barriers to fruit and vegetable consumption include cost, as well as limited availability and access (*6*–*8*). For some persons, such barriers might have worsened during the COVID-19 pandemic, related to economic and supply chain disruptions that could further limit ability to access healthier foods (*9*). Tailored intervention efforts to increase fruit and vegetable intake are needed to reduce age, sex, racial/ethnic, and income disparities in meeting fruit and vegetable intake recommendations among U.S. adults. States and communities can take actions by supporting food policy councils (community-based coalitions often supporting a specific community such as households with incomes below the federal poverty level or persons from racial and ethnic minority groups) to build a more sustainable food system,^†††^ supporting community retail programs to attract grocery stores and supermarkets to underserved communities to improve community food quality^§§§^ and increase healthy food access, promoting participation in federal nutrition assistance programs,^¶¶¶^ and implementing nutrition incentive and produce prescription programs**** that provide resources for persons to purchase fruits and vegetables. Additional efforts might include the use of nutrition standards, organizational food service guidelines,^††††^ and farm-to-institution approaches to ensure that culturally preferred fruit and vegetable offerings are available in work sites, hospitals, park and recreation centers, food banks and pantries, restaurants, and other locations (*10*). Education and social marketing can also help to ensure awareness of the recommended amounts of fruits and vegetables to consume and how to incorporate fruits and vegetables into meals and snacks.^§§§§^ Finally, conditions in which persons are born, live, learn, work, play, worship, and age, known as social determinants of health, affect health and influence the opportunities available to practice healthy behaviors. Ensuring that all persons, at all times, have physical, social, and economic access to enough foods, including fruits and vegetables that are safe, high quality, and meet their dietary needs and food preferences, requires multisectoral and multilevel collaboration.^¶¶¶¶^

The findings of this report are subject to at least five limitations. First, self-reported dietary behaviors are subject to recall and social desirability biases whereby different demographic groups might overestimate and others underestimate dietary intake.***** Second, BRFSS includes only noninstitutionalized adults; therefore, findings cannot be generalized to the entire U.S. adult population. In addition, U.S. territories were excluded because of the NHANES scoring algorithm. Third, using the algorithms to estimate intake might have resulted in measurement error. However, previous analyses showed that applying prediction equations to BRFSS frequency data yielded estimates comparable with national estimates that used more accurate 24-hour recalls (*4*). Fourth, 14% (59,589) of participants had missing fruit and vegetable data, and these respondents tended to be older and have a lower income. However, the percentage of missing data on fruit and vegetable and respondent characteristics are similar to that in previous studies (*4*,*5*). Finally, 16% (54,306) of participants had missing income data, but the estimated percentage of persons meeting recommendations was similar when missing income was imputed based on age, sex, and race/ethnicity.

Too few U.S. residents consume the recommended amounts of fruits and vegetables. Following a dietary pattern that includes sufficient fruits and vegetables can help protect against some chronic conditions that are among the leading causes of mortality in the United States (*2*); some of these conditions are also associated with more severe illness from COVID-19 (*3*). For most states, the BRFSS module is the only source of uniform, state-level dietary data for adults, and this information often provides critical metrics for state chronic disease plans. States can use the findings to guide their programs, communications and social marketing, and policies to support improving fruit and vegetable access and intake. Continued efforts to increase fruit and vegetable consumption by improving access and affordability in diverse community and institutional settings will help mitigate health disparities among U.S. residents.

**Summary**

**What is already known about this topic?**

The percentage of U.S. adults meeting fruit and vegetable intake recommendations is low.

**What is added by this report?**

In 2019, 12.3% and 10.0% of surveyed adults met fruit and vegetable intake recommendations, respectively. Meeting fruit intake recommendations was highest among Hispanic adults (16.4%) and lowest among males (10.1%). Meeting vegetable intake recommendations was highest among adults aged ≥51 years (12.5%) and lowest among adults with low income (6.8%).

**What are the implications for public health practice?**

States can use this information to tailor efforts to populations at high risk (e.g., men, young adults, and adults with lower income) and to implement enhanced interventions, policies, and programs that help persons increase fruit and vegetable consumption to support immune function and prevent chronic diseases.

## Footnotes

*
https://www.dietaryguidelines.gov/sites/default/files/2020-12/Dietary_Guidelines_for_Americans_2020-2025.pdf

† Appropriate for adults who engage in <30 minutes of moderate physical activity; more active adults might be able to consume more while staying within calorie needs. https://www.myplate.gov/eat-healthy/fruits; https://www.myplate.gov/eat-healthy/vegetables

§
https://www.healthypeople.gov/sites/default/files/NWS_ExecutiveSummary_2018-10.03.pdf

¶
https://www.cdc.gov/brfss/

**
https://www.cdc.gov/brfss/annual_data/2019/pdf/overview-2019-508.pdf

††
https://www.cdc.gov/brfss/annual_data/2019/pdf/2019-sdqr-508.pdf

§§
https://www.cdc.gov/nutrition/data-statistics/data-users-guide.html

¶¶ 45 C.F.R. part 46.102(l)(2), 21 C.F.R. part 56; 42 U.S.C. Sect. 241(d); 5 U.S.C. Sect. 552a; 44 U.S.C. Sect. 3501 et seq.

***
https://www.cdc.gov/nutrition/data-statistics/using-the-new-BRFSS-modules.html

†††
https://www.foodpolicynetworks.org/

§§§
https://www.cdc.gov/nccdphp/dnpao/state-local-programs/healthier-food-retail.html

¶¶¶
https://www.nal.usda.gov/legacy/fnic/usda-nutrition-assistance-programs

****
https://www.nutritionincentivehub.org/

††††
https://www.cdc.gov/nutrition/healthy-food-environments/food-serv-guide.html

§§§§
https://www.myplate.gov/eat-healthy/what-is-myplate

¶¶¶¶
https://www.cdc.gov/chronicdisease/programs-impact/sdoh.htm

*****
https://dietassessmentprimer.cancer.gov/concepts/
